# Effect of GH/IGF-1 on Bone Metabolism and Osteoporsosis

**DOI:** 10.1155/2014/235060

**Published:** 2014-07-23

**Authors:** Vittorio Locatelli, Vittorio E. Bianchi

**Affiliations:** ^1^Department of Health Sciences, School of Medicine, University of Milano Bicocca, Milan, Italy; ^2^Endocrinology Department, Area Vasta N. 1, Cagli, Italy

## Abstract

*Background*. Growth hormone (GH) and insulin-like growth factor (IGF-1) are fundamental in skeletal growth during puberty and bone health throughout life. GH increases tissue formation by acting directly and indirectly on target cells; IGF-1 is a critical mediator of bone growth. Clinical studies reporting the use of GH and IGF-1 in osteoporosis and fracture healing are outlined. *Methods*. A Pubmed search revealed 39 clinical studies reporting the effects of GH and IGF-1 administration on bone metabolism in osteopenic and osteoporotic human subjects and on bone healing in operated patients with normal GH secretion. Eighteen clinical studies considered the effect with GH treatment, fourteen studies reported the clinical effects with IGF-1 administration, and seven related to the GH/IGF-1 effect on bone healing. *Results*. Both GH and IGF-1 administration significantly increased bone resorption and bone formation in the most studies. GH/IGF-1 administration in patients with hip or tibial fractures resulted in increased bone healing, rapid clinical improvements. Some conflicting results were evidenced. *Conclusions*. GH and IGF-1 therapy has a significant anabolic effect. GH administration for the treatment of osteoporosis and bone fractures may greatly improve clinical outcome. GH interacts with sex steroids in the anabolic process. GH resistance process is considered.

## 1. Introduction

GH and IGF-1 are fundamental in achieving a normal longitudinal bone growth and mass during the postnatal period and, in association with sex steroids, play a major role in bone growth and development [1]. GH deficiency in childhood decreases bone mineral density (BMD), while GH treatment increases bone growth and strength [2]; IGF-1 is considered essential for longitudinal bone growth, skeletal maturation, and bone mass acquisition not only during growth but also in the maintenance of bone in adult life [3]. A positive correlation between serum IGF-1 level and BMD has been documented in women but not in men [4], in very old women [5], by others in men but not in women [6], and in healthy men [7]. Lower serum IGF-1 levels in women are reported to be correlated with the prevalence of fractures [8] and are strongly associated with an increased risk of osteoporotic fractures independently by BMD [9]. Recently, Ohlsson et al. [10] demonstrated that low serum IGF-1 levels were associated with an increased risk of fractures of about 40% and serum IGF-1 level could be clinically useful for assessing the risk of vertebral fractures [11]. Osteoporosis in postmenopausal women is due to estrogen (E) deficiency and having a high rate of bone remodeling with bone resorption exceeding bone formation [12]. The E deficiency is critical to the pathogenesis of osteoporosis in men and in women and the frequency of GH secretion is decreased in the amplitude with sex hormone reduction [13]. In young men the most significant hormonal determinants of the BMD of the hip and of the cortical thickness of the femoral neck are 17*β*-estradiol and IGF-1 while in aged men (over 60 years) the BMD was not correlated with IGF-1 at any site but only at 17*β*-estradiol [14]. It has been documented extensively that E is one of the factors regulating the expression of IGF-1 in maintaining skeletal integrity [15, 16]. The IGF-1 plays a central role in cellular growth, differentiation, survival, and cell cycle progression [17] and IGF-1 level is necessary for the proper acquisition of peak bone mass; furthermore GH protects against ovariectomy-induced bone loss [18]. Despite the evident anabolic effect of GH/IGF-1 on bone some conflicting clinical studies are evident. The aim of the present review is to evaluate the clinical studies published in the literature on the effect of GH and IGF-1 on bone metabolism in human subjects affected by severe osteoporosis and bone healing after surgery in non-GH deficient subjects.

## 2. The GH/IGF-1 Axis

GH is a polypeptide hormone secreted by the anterior pituitary gland. The synthesis of GH is under the control of central and peripheral signals. The primary site of GH action is the liver where it stimulates IGF-1 production [19]. GH has a direct effect on various tissues including liver, kidney, and muscle [20–22]. GH also acts on the central nervous system [23] and bone [24]. The action of GH is mediated by the binding of GH to the transmembrane GH receptor (GHR) which is present on the surface of most cells. The GHR is a major effector of human growth action and of the functional physiologic variants. GHRs in humans are expressed in the liver, adipose tissue, heart, kidney, intestine, lung, pancreas, cartilage, and skeletal muscle where they induce the synthesis of IGF-1 [25]. The GH has two different dependent and independent mechanisms of action, one directly through the GHR and the other inducing IGF-1 secretion. Circulating IGF-1 is mostly synthetized in the liver, but IGF-1 is expressed in all tissues, suggesting that autocrine/paracrine effects of local IGF-1 may be a major mechanism controlling tissue growth [26]. IGF-1 null mice exhibit severe growth retardation and mostly die soon after birth [27] although the autocrine/paracrine IGF-I, but not liver-derived IGF-I, is the major determinant of postnatal body growth [28] and the extrahepatic tissue levels of IGF-I are regulated by GH. IGF-1 circulates in a ternary form bound with the IGF binding protein IGFBP-3 and the acid labile subunit (ALS). There are six IGF-BPs and the IGFBP-3 represent the predominant binding protein for IGF-1 [29]. The IGF-1 regulatory system consists of IGF-1, IGF-II, and IGF-receptors and six binding proteins including IGFBP-1-6 [30]. Many central and peripheral factors regulate GH secretion [31]. IGFBP-3 is the principal binding protein in circulation, has similar affinity to both IGFs, binds 75% of IGFs in plasma [32], and regulates the free active IGF-1 concentration [33].

## 3. GH Intracellular Signaling

The GHR system utilizes the Janus kinase (JAK) signal transducer and activator of transcription (STAT) signal transduction pathway [34]. The activated GHR is associated with JAK2, a tyrosine kinase that once activated by GH, phosphorylates STATs-1, -3, -5a, and 5b tyrosine and Stat 5b is the predominant target of GH. The STAT proteins translocate to the nucleus where they bind to the specific DNA sequences and activate gene transcription. In addition, recent studies have indicated that suppression of cytokine signaling (SOCS) proteins also controls the GH signaling pathway [35]. These proteins play an important role in growth and skeletal development as well as in inflammation. In particular, the SOCS2 protein has a role in downregulating GH/IGF-1 signaling and has a functional role in the chondrocytes of the growth plate dynamics [36]. Chronic inflammation is associated with altered growth and skeletal development, and the SOCS proteins may also have an important role to play in mediating these effects.

## 4. Hormone Measurements

The valid determination of GH and IGF-1 in biological fluids is fundamental for a correct clinical evaluation [37]. Human GH is a heterogeneous protein hormone consisting of several isoforms and represents one important reason for the disparity among GH assay results from different laboratories [38]. Recently Frystyk et al. [39] discussed the current state of the art of IGF-I immunoassays and presented the analytical problems with IGF-I measurements. The pronounced binding of IGF-I to the high-affinity IGF-binding proteins (IGFBPs) constitutes a notorious source of error. For GH measurement generally is used Beckman Access Ultrasensitive human GH, a chemiluminescent assay with 96% specificity with 22 kDa human GH molecule and 4% cross reactivity with GH variant [40]. The serum IGF-1 is measured by the Immulite 2000 assay by Siemens Health care Diagnostic. The intra-assay for the serum IGF-1 ranges from 2.3 to 3.9% and the intra-assay CV range ranges from 3.7 to 8.1% [41]. For the detection of free hormones the mass spectrometry in the clinical laboratory has helped to develop proposed reference measurement procedures [42].

## 5. The Effect of GH/IGF-1 on Bone

The somatomedin hypothesis proposed by Salmon and Daughaday in 1957 [43] was that GH stimulates growth at the epiphysis by systemically derived liver IGF-1, but this has been challenged based on questionable direct effects of GH on chondrocytes* in vivo* and* in vitro* [44] and an alternative dual effector theory of GH action has been proposed [45].

*In vitro* studies using cultured chondrocytes have evidenced that GH stimulated the formation of colony of young precondrocytes directly while IGF-1 stimulated cells at later stage of maturation [46] and this preferential effect of GH on precondrocytes* in vivo* was also supported by another study of Ohlsson et al. [47]. GH is the major determinant of stimulation of progenitor cells and interacts with progenitor cells in adipose tissue and cartilage and IGF-1 stimulate a subsequent clonal expansion [48, 49]. GH and IGF-1 stimulate the preadipocytes at different stages of development and a direct action exerted on osteoblasts has been demonstrated [24]. The activity of IGF-1 on osteoblastic cells is increased by the presence of GH and IGFBP-3 [50]. IGF-1 is not able to substitute for GH in promoting this differentiation, but its mitogenic action selectively promotes cell multiplication in young differentiated clones.

GH and IGF-1 stimulate tissue growth with integrated functions. In fact IGF-1 is quite effective in stimulating growth in patients affected by GH insensitivity syndrome [51–55] but this effect becomes less effective due to lack of GH-induced IGFBP-3 stimulation of precondrocytes. Both GH and IGF-1 are considered potential anabolic agents because they play physiological roles in bone mass acquisition and maintenance [56]. The administration of GH and IGF-1 has the capacity to stimulate the longitudinal bone growth in animals and humans by acting both directly [57] and indirectly increasing local production of IGF-1 stimulating IGF-1-gene [51, 52, 58–61]. GH and IGF-1 have independent and different functions [62] and when the two compounds are given together they exert a synergistic effect [63].

### 5.1. *In Vivo* Studies

GH and IGF-1 have independent and different functions: the administration of GH to animals treated with maximal doses of IGF-1 stimulated growth further [62]. The effect was seen both in the tibia and in the femur whereas no significant effect was seen in the vertebrae. The lack of GH secretion and consequently low level of IGF-1 display osteopenia and reduced cortical bone, but normal trabecular bone in transgenic mice carrying a mutation of the GHRH receptor has been shown. Bone turnover was significantly reduced in GHR(−/−) mice, indicating GH involvement in the high bone-turnover level during growth. IGF-I treatment almost completely rescued all effects of the GHR(−/−) on both bone growth and remodeling, supporting a direct effect on both osteoblasts and chondrocytes [64]. IGF-1 plays an essential role in the development of the growing skeleton by influencing both longitudinal and transverse bone accrual and in the maintenance of bone mass during late adulthood and aging [65]. The administration of IGF-1 to GH-deficient animals and humans showed that both hormones have the capacity to stimulate longitudinal bone growth [53]. IGF-1-deficient mice exhibited skeletal malformations, a delayed mineralization, reduced chondrocyte proliferation, and increased chondrocyte apoptosis [66]. In mice carrying liver-specific IGF-1 deletion, which display a reduction in serum IGF-1, a decreased cortical bone is demonstrated [67]. The systemic IGF-1 contributes to cortical bone integrity, while the bone trabecular integrity is sustained by the locally produced skeletal IGF-1 [68]. A threshold concentration of circulating IGF-1 is necessary for normal bone growth and IGF-1, IGFBP-3, and ALS play a prominent role in the pathophysiology of osteoporosis [69].

### 5.2. GH and Bone Resorption* In Vitro*

Unlike bone formation, GH seems to have a more profound independent stimulatory effect on bone resorption. Proinflammatory cytokines such as TNF-*α*, IL-1beta, and IL-6 can promote osteoclastogenesis, and GH and IGF-1 can stimulate production of these cytokines in osteoblasts [70, 71]. Diminished GH receptor mRNA concentrations in response to IL-1 beta and TNF-alpha indicate that low IGF-1 levels during severe illness, despite high circulating GH levels, may at least partially be a consequence of suppression of hepatic GH receptor synthesis by IL-1 beta and TNF-alpha [72, 73]. GH increases IL-6 produced by human osteoblast-like cells. This* in vitro* evidence suggests, for the first time, a mechanistic paradigm by which GH modulates bone resorption [73]. The levels of GH, IGF-1, and some IGF binding proteins (IGFBPs) decrease in the elderly [5] and in osteoporotic subjects [74, 75] and low circulating levels of IGF-1 in elderly women are associated with greater femoral bone loss [76] suggesting a consistent effect of the anabolic IGF components on overall bone formation rate. Interestingly, the age-dependent attenuation of GH, IGF-I, and IGFBP-3 levels among healthy men, however, is not correlated with the reduction of BMD [4, 77] suggesting other hormonal interactions.

### 5.3. Role of IGFBP-3

The IGFBP-3 functions are complex and well summarized in a review by Yamada and Lee [78]. Current research demonstrating IGFBP-3's IGF independent roles in suppressing tumor formation and carcinoma cell growth has used autocrine/paracrine models [79, 80]. IGFBP-3 is able to modulate cell growth, the interplay between IGFBP-3 systemic and local regulation. The IGF-I/IGFBP-3 ratio, are positively related with PINP (N-terminal propeptide of human procollagen type I, a bone formation marker) and CTX (carboxy-terminal collagen crosslinks, a bone resorption marker) as a bone resorption marker in healthy adult men younger than 55 years and premenopausal women. In older subjects the found positive as well as negative relations with BTMs have to be further investigated [81].

## 6. Sex Steroids and Bone

E and androgens (A) exert potent influences on the size and shape of the skeleton during growth and contribute to skeletal homeostasis during adulthood [82]. Sex steroid hormones act on their target cells by binding to members of the nuclear hormone receptor superfamily: E binds to estrogen receptor ER*α* or ER*β*, and A bind to the androgen receptor (AR) [83]. Mouse models with cell-specific deletion of the estrogen receptors ER*α*, ER*β*, and the AR have provided novel insights into the function and signalling of these receptors on bone health and disease well evidenced by Monalagas et al. [84]. Human bone cells obtained from men and women have similar concentrations of AR and ER suggesting that both sex steroids have an important role in bone mass maintenance [85]. The A action on bone is more complex in males, because it not only activates the AR but also acts on two different estrogen receptors: receptor-*α* (ER*α*) or receptor-*β* (ER*β*) following aromatization, the conversion of T to E by aromatase [86]. The AR function is essential for normal bone growth and remodeling in male mice [87]. In mice completely lacking AR, a reduction in trabecular and cortical bone mass was observed [88]. A stimulate radial bone expansion in males and an optimal radial cortical bone expansion appears to require both AR and ER*α* signaling. The effects of E/ER*α* are mediated, at least in part, via interactions with the IGF system [83, 84, 89]. Ovariectomy and orchidectomy cause a dramatic increase in osteoblast and osteocytes apoptosis in mice. In addition, estrogens and androgens suppress osteoblast and osteocyte apoptosis induced by a variety of proapoptotic stimuli* in vitro* [90]. The effects of sex steroids on antiapoptosis are mediated via the classical ERs (ER*α* or ER*β*) or the AR. The distribution of these bone receptors on bone cells are at similar level in men and women [91] and this can explain the paradox why E restores bone mass in males with aromatase deficiency [92], while nonaromatizable androgens can protect the female skeleton against the adverse effects of estrogen deficiency [93]. An increase in osteocyte apoptosis following loss of estrogen in rats as well as in humans has been observed [94] and the accumulation of apoptotic osteocytes could increase bone fragility even before significant loss of bone mass because of the impaired detection of microdamage and repair of substandard bone. Activation of ER*α* has no effect on cortical and trabecular bone mass, which represents 80% of skeleton mass, indicating that the mechanism by which estrogen protects against the resorption of trabecular bone is on the osteoclasts [95]. The ER*α* is required for the osteogenic response to mechanical loading in a ligand-independent manner. Loading increases cortical bone area as a result of increased periosteal bone formation in both estrogen-sufficient and estrogen-deficient mice [96]. The AR has an important role in the homeostasis of the male skeleton with idiopathic hypogonadotropic hypogonadism or complete androgen insensitivity syndrome because of a loss-of-function mutation in AR having low bone mass [97]. The deletion of AR in male mice results in high bone turnover with a decreased trabecular and cortical bone volume [98]. AR is responsible for the preservation of trabecular bone in male mice [99].

## 7. Interaction of GH/IGF-1 with Estrogen

E is the major hormonal regulator of bone metabolism in both women and men [100] and is important for maintaining bone formation at the cellular level. E deficiency is highly correlated with bone resorption and has a major role in regulating bone resorption in men [101]. Falahati-Nini studied the relative contributions of testosterone versus estrogen in regulating bone metabolism in men. They observed that E accounted for 70% or more of the total effect of sex steroids on bone resorption in older men, whereas testosterone could account for no more than 30% of the effect, but androgen plays a fundamental role. The administration of GH in ovariectomized (OVX) rats increases cortical bone mass by inducing subperiosteal bone formation [102] resulting in an increase in bone volume, mineralizing surface, and osteoid surface. The effect of IGF-1 on the osteoblast progenitor cells is impaired in OVX mice [103]. These studies, using ovariectomized rats, indicate that GH alone stimulates bone growth and mineralization in the absence of estrogen alone. The association of GH plus E treatment in OVX rats resulted in an additive increase in the cancellous bone mass, which can be attributed to the suppressive effect of E on bone resorption and the anabolic effect of GH on bone formation [104]. GH and E reduced cancellous osteopenia, through different mechanisms. GH reduced the decrease in trabecular thickness, whereas E reduced the decrease in trabecular number and the increase in trabecular separation [105]. These results emphasize that not only bone loss is evident with E deficiency, but it is preventable by E administration. However, the administration of E is not able to restore bone loss [106]. High doses of E have a lowering effect on the serum level of IGF-1, and, in the proximal tibial metaphysis, E greatly suppresses the longitudinal growth rate and a lowered resorption of the cancellous bone mass [107]. The administration of E, but not T, significantly reduce circulating free IGF-I availability [108, 109] and the treatment with estrogen fails to restore the attributes of GH/IGF-1 axis in postmenopausal women. Conversely, low doses have a stimulatory effect on GH secretion in both sexes [110]. So that the association between bone mass and GH/IGF-1 level in women and not in men is due due to E inhibition on IGF-1 secretion, while free T induces an elevation. These effects may contribute to the gender differences observed in the GH-IGF-1 axis in healthy adults as well as in the responsiveness of hypopituitary patients to GH substitution [111].

## 8. Interaction of GH/IGF-1 with Androgens

A, including testosterone and its derivatives, are needed for skeletal growth and bone accrual during puberty and the effects are well evidenced in other reviews [112]. A stimulate radial bone expansion in males and an optimal radial cortical bone expansion appears to require both AR and ER*α* signaling, with the effects of estrogen/ER*α* being mediated, at least in part, via interactions with the IGF system [89]. A stimulate human and murine osteoblastic cell proliferation* in vitro*, and induce expression of the osteoblast-line differentiation marker ALP [113]. Oxandrolone stimulates production of osteoblast differentiation affecting the osteoblast AR and stimulating type I collagen synthesis [114]. The major role for AR activation in normal development is the trabecular bone and periosteal bone growth in male mice. Moreover, optimal stimulation of periosteal growth is only obtained in the presence of both AR and ER activation [115]. When GH and T were combined, the cortical bone area, periosteal bone formation rate (BFR), and femoral BMD were all significantly higher than that of the ORX and even higher than in the intact control rats. The treatment of androgen-deficient aged male rats by the administration of GH in association with T may have an independent effect in preventing osteopenia. The significant effect of GH+T may be attributed to the prevention of intracortical porosis and an increase in periosteal bone formation and cortical bone mass [116]. In humans, T indirectly stimulates IGF-1 secretion after aromatization into estrogen [117], while there is no evidence of fluctuation in GH responsiveness in hypogonadal men replaced with T or DHT alone [118]. Furthermore, aromatase inhibitors, that block the E formation from T, lower serum IGF-1 levels [119]. The predominating effect of androgens on IGF-I production can be explained by the tissue conversion of T to E. Long-term androgen deficiency results in a decrease in the calcium content of both tibia and lumbar vertebrae. In ORX old male rats the cancellous bone volume in the proximal tibial metaphysis was reduced by 50% four months after orchidectomy. At the same time, cortical bone was lost, the femoral cortical thickness was reduced by 12%, and cortical density tended to be lower. T, Dihydrotestosterone (DHT), E, or Nandrolone treatment completely prevented this decrease in cortical thickness and density. T and Nandrolone were also able to prevent the cancellous bone loss [120]. The administration of aromatizable and nonaromatizable androgens in association with GH therapy could be greatly effective to increase bone formation rate and mass. E is a potent inhibitor of bone resorption, whereas testosterone plays a synergistic role with GH in bone development and mass and the larger cortical bone size in males compared with females [115]. In pre- and postmenopausal women, the relative importance of A for bone health is increased [121]. In hypopituitary women A levels are low and free testosterone correlates with IGF-I. Discontinuation of E replacement in these patients induces elevations in IGF-I as well as free testosterone, and Delta IGF-I correlated positively with Deltafree testosterone. These effects may contribute to the gender differences observed in the GH-IGF-1 axis in healthy adults as well as in the responsiveness of hypopituitary patients to GH substitution [111]; a certain threshold level of testosterone is necessary to permit IGF-I stimulation. Administration of testosterone increases serum IGF-I levels in normal [122] and hypopituitary men [123, 124] and intact AR function is required for the suppressive effects of androgens on the osteoclastogenesis supporting activity of osteoblasts, but not osteoclasts [98, 125].

## 9. Effect of GH/IGF-1 on Osteoblasts and Osteoclasts

Bone turnover is the result of a balance between bone resorption and formation. The resorption process starts with recruitment of osteoclasts followed by the activity of osteoblasts by removing old bone and replacing it with a young matrix. Osteoblastic cells (osteoprogenitor cells) originate from a group of skeletal stem cells with osteogenic differentiation potential, referred to as skeletal mesenchymal stem cells (MSC). MSC reach bone surfaces from the circulation through vascular channels in association with bone remodeling sites [126]. Once they have arrived at the bone surface, osteoblastic cells produce bone matrix that becomes mineralized. The old osteoblasts die by apoptosis or become embedded in bone matrix as osteocytes. Osteoblasts and chondrocytes have receptors for GH and the administration of GH at physiological doses exerts a direct action on osteoblasts, stimulating cell proliferation and differentiation [24, 127]. There is a normal osteoblastic and osteoclastic response to GH also in osteopenic postmenopausal women [128]. IGF-1 reduces osteoblast apoptosis and promotes osteoblastogenesis by stabilizing *β*-catenin, enhancing Wnt-dependent activity [129]. The Wnt (Wingless and INT-1) family of signaling proteins influences most aspects of embryonic development and postembryonic tissue homeostasis [130]. Cellular responses to these proteins are often categorized based on their utilization of *β*-catenin, activity of the Wnt/*β*-catenin (“canonical”) pathway maintains transcriptional programs that enable stem cells to remain multipotent [131]. Osteoblasts exhibit complex Wnt-induced effects and Wnt is essential for osteoblastogenesis. For details see the review [132]. Also E and androgen have important effects on osteoblast life and function. E has been shown to inhibit osteoblast apoptosis and increases osteoblast lifespan [90]. Various studies have demonstrated that osteoblasts are directly stimulated by androgen [113, 133]. DHT (an androgen not convertible in estradiol) stimulates* in vitro* mineralization by a different mechanism from that of 1,25 (OH)D3 and TGF-*β* and also increases androgen receptors. Less clear is the function of IGF-1 on osteoclasts. Osteoclasts express IGF-1 receptors and IGF-1 has direct effects on their function [134].* In vitro*, IGF-1 induces RANK-L (receptor activator of nuclear factor *κ*B) synthesis and, as a consequence, osteoclastogenesis [135]. The induction of RANK-L by IGF-1 may explain the stimulatory effects of IGF-1 on bone resorption, whereas the induction of osteoprotegerin by GH may temper these effects [136].

### 9.1. IGF-1 Production by Osteoblasts

IGF-1 is produced locally by osteoblasts under control not only by GH, but also by other factors. Bone IGF-1 activity can be regulated at multiple levels [137]. It is well known that, in Ob cell models, both parathyroid hormone (PTH) and prostaglandin 2 (PGE2) can upregulate IGF-1 mRNA levels and that cAMP serves as the intracellular second signal [138]. Transient treatment stimulates collagen synthesis and the stimulatory effect is mediated by local production of IGF-I [139, 140]. The intermittent PTH treatment enhances osteoblasts differentiation through an IGF-I dependent mechanism and continuous PTH treatment enhances osteoclastogenesis through reciprocal which increases in RANKL and decreases in osteoprogerin [141]. The diverse effects on osteoblasts differentiation depending on the exposure time* in vitro* mediated through different signal transduction systems [142]. With aging there is an impaired osteoblastic function with reduced bone formation [143]. The role of GH/IGF-1/IGFBP signaling in age-related bone loss is very important: they promote osteoblastic cell proliferation and differentiation [127, 144] and the effects on osteoblasts and bone formation [145].

## 10. Mechanostat and Aging

There is inevitable age-related decline in bone mass that reflects a compromised signal transduction pathway that attenuates the ability of the skeleton to respond to osteogenic stimuli [146]. The physical stimuli are potent osteogenic signals in the young adult skeleton but are hardly evidenced in older bone tissue. The bone mass decline can be slowed or reversed by several therapeutic strategies: first, the effects of exercise and nutrition on the mechanostat model are fundamental [147] and represent the basic environmental factors known to affect muscle and bone development. Nutrition deficiencies have the most profound effect on mechanostat, and dietary protein is essential for bone health [148]. Mechanical forces are necessary to deform bone and stimulate bone regeneration and the trabecular tissue responds to diverse bone stressing exercises [149], although this is not consistent in all studies [150, 151]. Nutrition in association with hormonal factors works synergistically with exercise with respect to bone formation [152]. Aging processes could compromise signal transduction pathways reducing the tissue response to mechanical stimuli so that hormonal supplementation could be necessary.

## 11. GH and IGF-1 in Osteoporosis

The levels of GH, IGF-1, and some IGF binding proteins (IGF-BPs) decrease in the elderly [5] and in osteoporotic subjects [74, 75] and low circulating levels of IGF-1 in elderly women are associated with greater femoral bone loss [76] suggesting a consistent effect of the anabolic IGF components on overall bone formation rate. Serum IGF-I decreased with increasing age in both men and women and was higher in young women compared with young men in both cohorts, while the opposite was found in the highest age group [5, 6, 153]. Interestingly, the age-dependent attenuation of GH, IGF-I, and IGFBP-3 levels among healthy men, however, is not correlated with the reduction of BMD [77] suggesting that androgens interact with bone metabolism differently. In elderly men, BMD was not correlated with the IGF-I concentration [4]. In women with postmenopausal osteoporosis, serum IGF-I and II did not differ from the concentrations in normal women of similar age and did not correlate with BMD. There was no evidence that impaired synthesis of IGF-I and II contributes to the accelerated loss of trabecular bone and vertebral compression fractures [154]. The potential use of GH or IGF-1 as therapy for age-related bone loss is, however, controversial given their stimulatory effect on bone resorption, which may compromise their positive effect on bone formation. The complex mechanism of the effect of GH and IGF-1 on bone metabolism is evidenced in various clinical conditions that determine osteoporosis such as glucocorticoid induced osteoporosis, acromegaly, GHD patients, and senile osteoporosis.

States of GH resistance, such those observed in Laron dwarf patients, are characterized by mutations in the GHR resulting in a decreased serum and tissue IGF-1 levels. The IGF-1 replacement therapy in such patients increases growth velocity but does not normalize growth. In the GH-resistant mice (GHRKO-HIT mice) [155, 156], serum IGF-1 levels were restored via transgenic expression of Igf-1 and normalization of serum IGF-1 improved body adiposity and restored glucose tolerance but was insufficient to support normal skeletal growth, resulting in an osteopenic skeletal phenotype. The inability of serum IGF-1 to restore skeletal integrity in the total absence of GHR showed that GH-mediated effects on the skeleton are independent of serum or tissue IGF-1. These findings showed that IGF-I replacement therapy in patients with Laron syndrome does not achieve full skeletal growth [156].

### 11.1. Glucocorticoid Induced Osteoporosis

Glucocorticoid treatment causes osteoporosis and growth retardation in humans. Chronic glucocorticoid treatment induces a redistribution of spontaneous PTH secretory dynamics by reducing the amount released in tonic fashion and increasing the amount released as pulses [157]. Manolagas and Weinstein [158] found that in rodents as well as in humans increased apoptosis of osteoblast and osteocytes is a key pathogenetic mechanism of the adverse effects of glucocorticoid excess on bone. GH and IGF-I, but not GH alone, markedly counteracts diminished bone and body collagen synthesis caused by glucocorticoids, whereas connective tissue resorption is enhanced during treatment with GH alone or in combination with IGF-I [159].

### 11.2. Acromegaly

Acromegaly is caused by a pituitary tumor that secretes GH and the disease is characterized by elevated plasma levels of GH and IGF-1 [160] and their effects on bone mass and strength are not yet clearly defined. In acromegaly, GH and IGF-1 excess increases bone turnover and remodeling [161]. The serum concentration of osteocalcin is positively correlated with GH and IGF-1 levels [162]. The most studies reported an increase in BMD at the femoral neck, gender-independent [163–166]. Other studies showed a normal BMD [167] despite hypogonadism [168]. The normal BMD at lumbar spine was found in various studies [163, 164, 166–170]. Zgliczynski et al. [171] found a smaller increase in bone resorption and greater increase in BMD in women with acromegaly than in men. Vestergaard and Mosekilde [172] found that fracture risk was significantly decreased in patients with acromegaly compared to controls due to the anabolic effect of GH on bone. The anabolic effect of GH on trabecular and cortical bone remained demonstrable after remission of acromegaly; the mean BMD remained normal both at lumbar spine and femoral neck but not at cortical sites in the long term [173]. In contrast, the bone histomorphometry by iliac crest bone biopsies demonstrated a significant increase in bone turnover with a decrease in cortical and trabecular bone mass [174, 175]. The first observation that acromegaly could be a cause of osteoporosis was made by Riggs et al. [176] and more recently it has been demonstrated that acromegalic patients could be at risk of osteoporosis and fractures [177] and vertebral fractures [178]. Following studies clearly demonstrated that trabecular structure of bone is abnormal in relation to biomechanical properties in acromegalic patients compared to controls [178, 179] and these patients were at high risk of vertebral fractures, confirming previous observation of an unfavorable effect of chronic excess of GH on the skeleton. For the first time using quantitative morphometric analysis (a 3D HR-pQCT system) and BMD at lumbar spine and total hip, Bonadonna et al. [180] and Mazziotti et al. [136] found a high prevalence of osteoporotic vertebral fractures in unselected acromegalic patients. This hypothesis, already based on association studies, has been recently confirmed by prospective studies [181, 182]. Mazziotti et al. after a 3-year follow-up demonstrated a high rate of incident vertebral fractures both in patients with active and controlled acromegaly (42.0%) compared to controls subjects (3.8%). Claessen et al. [182] showed that vertebral fractures progress in the long term in 20% of patients with biochemically controlled acromegaly in the absence of osteoporosis or osteopenia suggesting that an abnormal bone quality persists in these patients after remission, possibly related to pretreatment long-term exposure to high circulating levels of GH. Vertebral fractures may also develop even in patients with normal BMD [180, 183, 184]. Bonadonna et al. [180] showed that high numbers of postmenopausal women with acromegaly develop vertebral fractures which occurred even in the presence of normal BMD, whereas, in patients with controlled acromegaly, vertebral fractures were always accompanied by a pathological BMD. In both diabetic and nondiabetic patients, vertebral fractures occurred independently of BMD and suggest that diabetes mellitus may be associated with an increased prevalence of vertebral fractures in males with acromegaly [183]. Acromegalic patients with fractures had significantly higher serum IGF-I values. This effect seems to be relatively attenuated in the presence of persistent GH hypersecretion. Wassenaar et al. [184] found that BMD did not change during prolongation of follow-up by 7 years of controlled acromegaly and the high prevalence of vertebral fractures were independent from BMD. The biochemical control of acromegaly may not be sufficient to normalize the risk of vertebral fractures mainly in patients with hypogonadism. The association of the incidence of vertebral fractures with hypogonadism was significantly higher in patients with active disease [181]. Acromegaly appears to have a deleterious effect on trabecular bone microarchitecture and the gonadal status might be more important than type 2 diabetes mellitus or acromegaly activity in determining bone health [178]. Moreover, patients with fractures showed significantly longer untreated hypogonadism as compared to patients without fractures. The GH/IGF-1 hypersecretion may produce different effects on bone in males and females under the influence of estrogen/testosterone and age [171]. The effect of chronic GH excess on spinal trabecular bone mass seems to be anabolic in active eugonadal patients but not in hypogonadal patients [185]. These data show that although high levels of circulating GH and IGF-1 have an anabolic effect on bone, they alone are not able to maintain BMD and the disadvantageous effect on bone density was associated with hypogonadism in both sexes [171, 185]. Sex hormones have a crucial role in maintaining the trabecular bone microstructure; the deleterious effects of hypoganadism on bone microstructure have been described in postmenopausal women [186–188] and in men [189] with reduced cortical bone and lower trabecular density and thickness. These data underscore the important interaction of sex steroids with GH/IGF-1 axis on bone metabolism.

### 11.3. Treatment of Growth Hormone Deficiency (GHD) Patients

GH treatment in GHD adults has a marked effect on markers of bone formation (such as osteocalcin and serum level of C terminal propeptide), bone resorption (urinary hydroxyproline, collagen cross links, and serum concentration of collagen type I telopeptide), and serum IGF-1 levels [190–192]. Trials involving adults or childhood-onset GHD show conflicting results regarding the effect of GH on bone mass. However, short-term placebo controlled trials have failed to show any increase in BMD or BMC during GH treatment [190, 192–194]. More encouraging results were obtained after more prolonged treatments (12–30 months) [195–198]. In the latter study, 10 years of GH replacement was conducted in hypopituitary adults and a sustained, progressive increase in bone mass and bone density was observed. The study also suggests that adequate estrogen replacement is needed in order to have an optimal response in BMD among GHD women. Elborsson et al. [199], using data from GHD adults with fifteen years of GH replacement with a low dose of 0.6 mg/day, demonstrated a sustained increase in total body and lumbar spine BMC and BMD. In the neck of the femur, BMC and BMD peaked at 7 years and then decreased toward baseline values. Lumbar spine BMC increased by 9% and BMD by 5% while in the femur neck the increase in BMC and BMD was 7% and 3%, respectively. After 15 years, femur neck BMC was 5% above baseline. Gender differences have been observed in response to GH. Males responded with a high increase in serum osteocalcin, PICP, and CITP concentration, whereas in women a more marked increase in total body BMD was observed [200]. A cross-sectional study reported that vertebral fractures were significantly more frequent in GHD patients versus control subjects and the fracture prevalence, as well as the fracture number, was significantly higher in untreated versus treated patients. The prevalence of spinal deformities was correlated only with the timing of the beginning of rhGH replacement and not with BMD. The replacement treatment of GHD leads to a significant decrease in fracture rate [201]: recently, the meta-analysis of Barake et al. [202] suggests a beneficial effect of rhGH replacement on BMD in adults with GH deficiency mentioning that larger studies are needed to evaluate the effect of rhGH on fracture risk.

### 11.4. Senile Osteoporosis

In elderly people, the bone mass declines and the risk of fractures increases [203]. Fragility fractures and impaired or unsuccessful bone healing are the most significant consequences of osteoporosis [204]. Several interacting factors contribute to the risk of osteoporosis, including clinical, medical, behavioral, nutritional, hormonal, and genetic variables [205, 206]. Sarcopenia and other muscular factors also contribute to bone loss and increased fracture risk in the elderly [207] associated with physical impairment and functional limitation [208]. Both trabecular and periosteal bone formations decline with aging in males and females [209]. The key feature of osteoporosis in postmenopausal women and in aging men was identified in estrogen deficiency. Estrogen plays a dominant multifactorial role in maintaining cortical bone formation by supporting osteoblasts and preventing bone resorption by suppressing osteoclast formation and stimulating osteoclast apoptosis [210]. In the first years after menopause, bone loss accelerates [211] and bone loss continues with the increasing age [212]. In men osteoporosis sustains approximately 30% of all hip fractures and has a 2-fold excess mortality within the immediate postfracture period [213]. Men also suffer greater functional impairment from severe vertebral deformities compared to women [214] suggesting that factors other than BMD are also important in determining risk [215]. Manolagas [216] provided a paradigm shift from the “estrogen-centric” role of the pathogenesis of osteoporosis to an intrinsic oxidative stress age-related in organs and tissues. The oxidative stress in both animals and humans may be a pivotal pathogenetic mechanism of the age-related bone loss and strength. Loss of estrogens or androgens accelerates the effects of aging on bone [16]. The late phase of bone loss in aging women and men is mediated by the increased PTH levels [217]. Aging is associated with a decrease in GH secretion [218] and serum IGF-1 concentration has been reported to be significantly lower in men [219] and women with osteoporosis. Positive relationships between BMD and serum concentrations of IGF-1and IGFBP-3 were observed in healthy men [220]. The circulating level of IGF-1 was found to be an independent predictor of total BMC in healthy elderly women [221] and was significantly lower in men [219] and women with osteoporosis. In addition, IGFBP-3 has been reported to be lower in both males and females with osteoporosis [222]. The age-related increase in PTH secretion was due to the loss of the effects of estrogen on extraskeletal calcium homeostasis and this action seems to be more dominant even in elderly women. Also important to note is that vitamin D deficiency with aging contributes independently to secondary hyperparathyroidism [223]. Aging is also associated with decreases in the amplitude and frequency of GH secretion [13]; sex and age have independent and interrelated effects on GH secretion. These effects can be largely accounted for by corresponding variations in endogenous estradiol levels amplifying the action on the neuroendocrine regulation of pulsatile GH release [18]. In GHD patients, serum IGF-1 levels are low in females and males, and when GH therapy is given, females require a higher dose of GH to get an increase in serum IGF-1 levels similar to males [224] evidencing a GH resistance to exogenous or endogenous E. Furthermore, circulating IGF-1 is positively associated with aerobic fitness and muscular endurance and in young men is an expression of health and fitness outcomes [225–227]. Nutrition is another important factor regulating IGF-1 secretion. Changes in free IGF-I and IGFBP-1 are sensitive to caloric restriction [228] and a positive correlation between protein ingestion and bone health has been found [229, 230]. Finally, the aging process is associated with increased serum TNF*α* levels [231]. This cytokine inhibits bone formation in part by inducing osteoblast apoptosis [232] and the TNF*α* antagonists have been found to reverse the age-related deficit in bone formation [233].

## 12. Effect of the GH/IGF-1 Axis on Bone Metabolism in Patients with Normal GH Secretion

The GH/IGF-1 axis is one of the major determinants of adult bone mass [234]. It has been well established that GH secretion declines with age [235] and IGF-1 declines in serum and bone [236]. GH and IGF-1 are of primary importance for bone mass in addition to IGFBP-3. A positive relationship between BMD and serum concentrations of IGF-1 and IGFBP-3 was observed in healthy men [7] and rapid decreases in serum IGF-1 levels after menopause might be partly involved in bone loss [237]. Various cross-sectional studies have demonstrated strong relationships between serums IGF-1 and IGFBP-3 and BMD in postmenopausal women and men [238–240]. In young men, with age under 60 years, the IGF-1 level was a determinant of hip BMD (Szule P, 2004) and low serum IGF-1 levels are associated with increased risk of hip and vertebral fractures by 45% and 40%, respectively [10]. In elderly women, serum IGF-1 concentration was found to be an independent predictor of total BMC [221] and low levels of IGF-1 have been reported in both males and females with osteoporosis [222].

## 13. Effect of GH Administration on Osteoporosis and Bone Metabolism

The administration of GH has been evaluated in various clinical trials in normal subject and in osteoporosis and postmenopausal women (Table 1). A great deal of variance exists in the studies in terms of age, dose of GH administered, and duration of the therapy. Studies have reported large differences in age (from 22 to 81 years) and in the administered dose of GH (from 0.015 mg/kg/day to 0.75 mg/kg/day) in addition to the duration of the therapy ranging from 3 days to 3 years. In a large part of the studies a significant increase in bone formation, BMC, and BMD was observed. Firstly Kruse and Kuhlencordt in 1975 [241], in patients with primary and secondary osteoporosis treated with GH, reported an increase in periosteal new bone formation and an intracortical bone resorption with a significantly increased relative osteoblast activity. The effect of GH therapy has been evaluated in healthy subjects [242–245], in postmenopausal osteoporosis [128, 244, 246–256], and in men with idiopatic osteoporosis [254, 257]. In the most studies a significant bone resorption and bone formation with a prevalent anabolic effect and BMD increased were observed. Only in a few studies the evaluation of BMD by DEXA (Dual-energy X-ray absorptiometry, a mean of measuring BMD) was described [240, 252, 256]. The Landin-Wilhelmsen [255] the longest double blind, randomized placebo-controlled trial, showed conducted for three years, an increase in bone mineral content of 14% in postmenopausal women with osteoporosis. In a few studies no positive effect of the GH therapy was observed [244, 246, 248, 252]. In the Saaf study the GH dose administration was reduced by 50% due to side effects and after one year a reduction of BMD at the femoral neck was observed. The reason for this apparent partial resistance to the anabolic effects of GH was not clear but nutritional deficits and the low doses of GH administration were suspected. In the Holloway study the dosage of GH administered was very low (0.02 mg/kg/day) as for the Erdtsieck study (0.02 mg/kg/day three times a week). GH administration also restores normal PTH secretory patterns in osteoporotic postmenopausal women, improves target organ sensitivity to PTH, and results in a net positive balance in bone mineral metabolism, all leading to an increase in BMD [256, 258]. The women receiving estrogen therapy showed a less increment of all bone markers [244] suggesting, in concordance with the data from Ho and Weissberger [259], that hepatic IGF-1 generation was suppressed due to oral delivery of estrogen. The most frequent side effects observed due to GH therapy were fluid retention and carpal tunnel syndrome which disappeared after withdrawal, but generally the therapy was well tolerated.

## 14. Effect of IGF-1 Administration on Osteoporosis and Bone Metabolism

Recombinant human IGF-1 (rhIGF-1) has been used for many years for the treatment of osteoporosis and the clinical studies are reported in Table 2. In clinical studies on this issue, it is evident that age has varied considerably (from 12 to 74 years), which may explain some differences in results. Furthermore, great differences in the dose have been reported (from 0.5 to 240 *μ*g/kg/day) and the duration of the therapy has varied from 1 to 6 days. Significant bone resorption and formation has been demonstrated in all studies and no significant side effects have been reported. An increase in circulating IGF-1, IGFBP-2 and IGFBP-3, and PINP (a bone formation marker) has also been reported. The studies have been performed in varying clinical conditions, including healthy subjects [245, 266, 268], during fasting [265], in osteopenia and anorexia [267, 271, 273], in postmenopausal osteoporosis [263, 269], in men with osteopenia [257, 262, 280], osteoporosis in Werner syndrome [264], and during corticoid therapy [159]. In all the studies a positive effect on bone remodeling and increased bone formation was evidenced. Only in one study after long-term IGF-1 administration for 1 year, no differences in bone turnover markers between the IGF-1 treated and placebo treated subjects were found [269]. The negative finding in this investigation could be attributed to the fall of GH secretion due to IGF-1 administration. Previous studies demonstrated that a low dose IGF-1 treatment increased blood markers for bone formation without increased for resorption markers in osteoporosis of postmenopausal women [263, 266] and a dose response effect was described. Long-term studies have shown similar positive effects and generally have shown a good safety record with low dosage recombinant human IGF-1 on bone mass. Grinspoon et al. [272] studied low weight, osteopenic girls with anorexia nervosa, and were the first to report that bone turnover falls rapidly with acute caloric deprivation in normal women and rhIGF-1 administration selectively stimulated bone formation in this setting.

Berneis et al. [159] demonstrated that only the combination of GH and IGF-I, but not GH alone, markedly counteracts the diminished bone and body collagen synthesis caused by glucocorticoids. The free testosterone index was lower in these patients. In conclusion it looks that short- and long-term treatment with IGF-1 stimulates bone resorption and bone formation considerably with prevalent anabolic effect and produced minimal or no side effects.

## 15. Effect of GH/IGF-1 on Fracture Healing

The complexity of the fracture repair process has been reported since its first histologic description. In a fractured bone the healing process is initiated by the formation of a thick periosteal callus of woven bone with a central area of cartilage. Endochondral ossification of the cartilage occurs subsequently in the woven bone. Later a marked remodeling process is activated and the callus volume declines while the density is enhanced. In epiphyseal plate after fracture the healing was accompanied by an increase in DNA content, by a change in cellular activity, and by greatly accelerated apoptosis [281]. Various cellular mediators (bone morphogenetic proteins, interleukins, and angiogenic growth factors) in healing bone are involved [282]. The potential for growth and differentiation factors, such as the use of BMPs to enhance fracture healing in the clinical setting is still controversial [283]. In tibiae of micropigs it has been demonstrated that callus area, bone area, cartilage area, and bone perimeter were regenerated after GH administration and that GH promotes bone formation and maturation of the regenerate without disturbing the callus structure [284].

Many experimental and clinical studies have found that growth factors, such as morphogenetic proteins (MPs) [285], the fibroblastic-like growth factors family (FGFs) [286], transforming growth factors (TGFs) [287], and plateled-derived growth factors (PDGF) [288], stimulate bone formation during fracture healing. However, these hormones require local application which leads to difficult administration due to limited accessibility to the fracture site. Evidences suggests that these hormones can also influence bone healing in patients who have sustained head injury with a more intensive callus formation than those without head injury [289, 290] and one injury to one part of the skeleton increases bone formation at a distal skeleton site [291] indicating that systemic humoral mechanisms may enhance bone formation. The GH/IGF-1 axis is involved in the biochemical mechanisms determining delayed or failed fracture healing [292]. The clinical trials conducted on the effect of administration of rhGH or rhIGF-1 on bone healing are reported in Table 3.

The studies evidenced a significant effect of GH treatment on bone healing in patients after surgery for hip or tibial fractures. The range of dose of administration varied from 0.02 to 0.06 mg/kg/day. Various studies have investigated the effect of GH therapy in hip fractures [274–277] all reporting a positive effect on bone healing and functional outcome. Two studies have investigated the effect of GH therapy on tibial fractures. In one study [279] dose dependent markers of bone formation were described and the biochemical markers of bone turnover persist until eight or twelve weeks after the cessation of treatment. Raschke et al. [278] performed a randomized double-blind placebo-controlled trial of GH treatment at different doses (15, 30, and 60 *μ*g/kg/day), in a group of open and closed tibial fractures, and reported no significant enhancement of fracture healing. The reason of this GH resistance could be related to a minimum increment in IGF-1 (from 201 to 218 ng/mL) and in IGFBP-3 (from 2.5 to 2.9 mg/L) and other factors could have interfered with the GH effect, such as nutritional and inflammatory status. Only one study reported the effect of IGF-1 on bone healing [270]. In this study a protein complex rhIGF-I/IGFBP-3 in the treatment of 20 women affected by hip fractures was used and indicated that this treatment at a dose of 1 mg/kg/d stimulates bone metabolism in frail osteoporotic patients and the muscular efficiency increased. Placebo-treated subjects had an average bone mineral loss at the contralateral hip of about 6% during the first 6 months after the injury. The IGF-1 serum level is significantly associated with the ability to function after hip fracture in women [293]. GH therapy is also helpful for prevention of fracture risk in GH deficient subjects [294]. In a recent review [295], the effectiveness of GH on hip fracture healing was evaluated and treatment showed that IGF-1 levels significantly increased in the short term, but no significant differences occur in the long term. Only one study measuring BMC showed no significant differences in the change of the BMD scores between GH and placebo groups and only one trial reported the measurement of BMC. The latter trial showed that GH had no effect while the placebo group lost BMC at both 4 and 8 weeks. With the low quality of current evidence, GH may be effective in hip fractures. More carefully designed, double-blinded and placebo-controlled randomized trials with large numbers of participants are needed to better evaluate the effects of GH in the treatment of the various fractures.

## 16. GH Resistance

The development of GH resistance is one of the most important metabolic derangements observed in patients with systemic infection, major trauma, and burn injuries [296, 297]. GH resistance probably has a multifactorial origin. The clinical consequences of GH resistance include weight loss (particularly lean body mass), impaired wound healing, prolonged recovery, and impaired survival [298]. It is likely that both the fasting state and circulating factors, possibly inflammatory cytokines, such as TNF and IL-1, are etiological factors [298–300]. Data from* in vitro* studies and animal models suggest that increased levels of inflammatory cytokines can induce cachexia and might inhibit the effects of GH on target tissues. During sepsis, growth hormone (GH) resistance contributes to the catabolism of muscle protein suggesting that TNF mediates hepatic GH resistance during sepsis by inhibiting the duration of signaling via the Janus kinase-2/STAT5 pathway [301]. It has been demonstrated that the inhibitory effects of interleukin-1 on growth hormone action during catabolic illness [298] and also Interleukine-6 inhibit growth hormone-mediated gene expression in hepatocytes [302], so the liver becomes unresponsive to growth hormone. Resistance or insensitivity is characterized by higher GH levels, low IGF-1 levels, and a reduced anabolic response to GH. This aspect must be considered before starting a therapeutic program with GH in patients after surgery or with an active inflammation process because GH resistance could inhibit GH effects.

## 17. Cancer Risk Related to GH and IGF-1 Administration

The GH and IGFs have mitogenic and proliferative properties and the potential risk in tumor promotion and progression has been suspected [303, 304], but various studies have demonstrated that this risk is uncertain. Deodati et al. [305] assessed systematically the association between GH therapy and all-cause, cancer and cardiovascular mortality, cancer morbidity, and risk of second neoplasm mainly in patients treated during childhood and adolescence. Malignancy and cardiovascular SMRs were not significantly increased. The overall cancer incidence and the relative risk for second neoplasms were significantly increased. Carel et al. [306] found that the mortality rates were increased in a population of adults treated from childhood age with recombinant GH, particularly in those who had received the highest doses. Specific effects were detected in terms of death due to bone tumors or cerebral hemorrhage but not for all cancers.

In a long-term mortality study none of the patients died from cancer or from a cardiovascular disease [307]. In GH-deficient adults treated with GH the occurrence of malignancies was not higher than in the general population [308]. In cancer survivors treated with GH the elevation of risk of developing a second neoplasm due to GH use appears to diminish with increasing length of follow-up [309]. Higher IGFBP-2 and/or IGFBP-3 may be associated with increased cancer risk [310], but a recent meta-analysis evidenced that the IGFBP-3 polymorphisms are not associated with colorectal cancer susceptibility [311]. In patients who received GH treatment after 24 weeks and 48 weeks side effects were small, with no significant differences between subgroups [312]. The data may raise concern on the long-term safety of GH treatment, but for short time therapy only minimal side effects, such as fluid retention or articular pain, were described.

## 18. Conclusions

GH and IGF-1 have a great effect on bone resorption and bone anabolism and their administration has a positive effect on osteoporosis and fracture healing. Androgen and estrogen interact with GH and IGF-1 optimizing bone mass acquisition with prevalent effect of androgen [89, 313]. Sex steroids are critical for skeletal growth and maintenance. While IGF-1 has a great effect on bone resorption at trabecular level, androgens play a fundamental role on bone activating directly and indirectly the AR increasing the trabecular and periosteal bone growth. The optimal stimulation is obtained in the presence of both AR and ER activation and a minimum for E level is necessary in men for bone maturation [314]. The effect of GH and IGF-1 therapy on bone osteoporosis and fracture healing is mediated by many factors that can realize a GH resistance: inflammatory cytokines, sex hormones levels, nutritional deficiency, and muscle mass and strength. More carefully designed, double-blind, and placebo-controlled randomized trials with large numbers of participants regarding GH plus sex hormone treatment of osteoporosis and bone healing after fractures are required.

## Figures and Tables

**Table 1:** Effect of GH administration on osteoporosis and bone metabolism.

Authors	Subjects and clinical conditions	Age years	Dose mg/kg/day	Duration	BF	BR	BMD	Side effect
Kruse and Kuhlencordt, 1975 [241]	3 MO	58, 36, 45	1.45 to 2.3 mg/day	8 to 15 months	↑	↑	New periosteal bone formation and osteoblasts significantly increased	Fluid retention and carpal tunnel syndrome

Brixen et al., 1990 [242]	20 M healthy	26.5 ± 5.6	0.06	7 days	↑	↑	BMC got significantly higher	None

Rudman et al., 1990 [243]	12 M healthy	72.1 ± 8.5	0.027 × 3 times/week	6 months	—	—	Increase in lumbar vertebral BMD and in lean body mass	None

Marcus et al.,1990 [260]	16 M and W	60	0.03, 0.06, or 0.12	7 days	↑	—	PTH and osteocalcin increased	Impaired oral glucose tolerance

Clemmesen et al., 1993 [246]	42 PMO	71.6 ± 3.0	7.2 mg/week	12 week	↑	↑	Bone mass decreased	None

Kassem et al., 1994 [247]	30 PMO	69 ± 5.6	0.067	3 days	↑	↑	Significantly increased serums IGF-I, IGF-II, IGFBP-3, and IGFBP-4	None

Holloway et al., 1994 [244]	19 W healthy	64.6 ± 2.9	0.02	6 months	↑	↑	No significant changes at the lumbar spine and femoral neck	Fluid retention and carpal tunnel syndrome

Brixen et al., 1995 [128]	40 PMO	52–73	0.015–0.03–0.06	7 days	↑	↑	Dose-dependent stimulation of bone formation and bone resorption	None

Erdtsieck et al., 1995 [248]	21 PMO	63.5 ± 9	0.020 3 times/week + pamidronate	1 year	↑	↑	Blunted the pamidronate induced accumulation of bone mineral mass and the reduction of bone turnover	None

Johansson et al., 1996 [257]	12 MIO	44 ± 8	0.60 mg/m^2^ or IGF-I (80 micrograms/kg)	7 days	↑	↑	IGF-I enhanced formation of collagen type I more than GH did	None

Bianda, 1997 [261]	7 M healthy	32 ± 6.4	3.63	5 days	↑	↑	Bone turnover and free calcitriol index increased	None

Holloway et al., 1997 [249]	84 PMO	69.2 ± 6.5	0.020 (7 days followed by 5 days of calcitonin 100 U)	2 years 12 × 56 days cycles	↑	↑	Significant increases of BMD at lumbar spine and hip in the combined GH + CT and GH + placebo	None

Kassem et al., 1998 [250]	40 PMO	52–73	0.015–0.03–0.6	7 days	↑	↑	Significantly increased serums IGF-I, IGF-II, IGFBP-3, and IGFBP-4	None

Sugimoto et al., 1999 [251]	8 F	71 ± 3.4	0.038/kg/week followed by 0.075/kg/week	4 weeks 48 weeks	↑	↑	Increases in radial and lumbar BMD, effect after discontinuation of GH treatment	None

Sääf et al., 1999 [252]	12 PMO	67.8 ± 1.1	0,015 and reduced to 50%	1 year	No change	No change	Decreased BMD at femoral neck	None

Sugimoto et al., 2002 [253]	8 PMO	72.0 ± 0.5	0.0054	48 weeks	↑	↑	Radial BMD significantly increased after discontinuation of GH treatment	None

Gillberg et al., 2002 [254]	29 M IO	47.8 ± 9.8	0.36	12 months	↑	↑	Increased BMD and BMC sustained for at least 1 yr after treatment	None

Landin-Wilhelmsen et al., 2003 [255]	80 PMO	50–70	0.30 or 0.75 mg/day + estrogen	3 years	↑	↑	BMC increased 14%	None

Joseph et al., 2008 [256]	14 PMO	63.4 ± 2.0	0.2 mg/d × 4 wk increments of 0.1 mg/d every 2 wk	12 months	↑	↑	Net, increase in BMD, and sensitivity to PTH restored	None

M = men, W = women, PMO = postmenopausal osteoporosis, MIO = men with idiopatic osteoporosis, BF = bone formation, BR = bone resorption, and BMD = bone mass density.

**Table 2:** Effect of IGF-1 administration on osteoporosis and bone metabolism.

Authors	*n*. subjects	Age	Dosage *μ*/kg/day	Duration	BF	BR	Clinical effects
Johansson et al., 1992 [262]	1 M	—	80 *μ*/kg/day × 2		↑	↑	Bone formation markers increased

Ebeling et al., 1993 [263]	18 PMO	74 ± 0.2 66.3 ± 7.8 67.4 ± 5.5 58.6 ± 4.4	30 60 120 180	7 days	↑	↑	Significant dose-dependent BR, less BF. Longer treatments are suggested

Rubin et al., 1994 [264]	1 W, Werner syndrome, low serum IGF-1 level, and osteoporosis.	—	30–75	6 months	↑	↑	Lumbar bone mass increased 3% BMD

Grinspoon et al., 1995 [265]	14 W normal	19–33	100 *μ* × 2	6 days	↑	—	Increase bone formation

Ghiron et al., 1995 [266]	16 w healthy	71.9 ± 1.3	60, 15 *μ* (diet also)	28 days	↑	↑	Increase bone mass

Johansson et al., 1996 [257]	12 M IO	44 ± 8	80 *µ*	7 days	↑	↑	Enhanced bone formation and bone resorption

Grinspoon et al., 1996 [267]	23 W	18–29	200, 60 *µ*	6 days	↑	↑	Increases markers of bone turnover

Mauras et al., 1996 [268]	5 M + 3 W 3 M + 2 W	23–27 23–25	240 *µ* 100 *µ*	5–7 days	↑	↑	Synergize with sex steroids to maximally stimulate attainment of peak bone mass in humans

Bianda et al., 1997 [245]	7 M		192 *µ*	5 days	↑	↑	Serum osteocalcin and PICP, the urinary deoxypyridinoline/creatinine, and calcium/creatinine ratios got significantly higher

Berneis et al., 1999 [159]	24 M healthy	24.5 ± 1.2	GH = 0.15 × 2 IU/kg/day + IGF-1 = 80 *µ*	6 days	—	—	Markedly counteracts diminished bone and body collagen synthesis caused by glucocorticoids

Friedlander et al., 2001 [269]	24 PMO	72 ± 2.7	15 *µ*/kg twice daily	1 year	↑	↑	No effect

Boonen et al., 2002 [270]	30 W	65–90	0.5–1 *µ*/kg/d	8 weeks	↑	↑	Effects on bone mass, muscle strength, and functional ability, beneficial trends

Grinspoon et al., 2002 [271]	60 W osteopenia with anorexia	18–38	30 *μ*g/kg sc twice daily	9 months	↑	—	Increased mIGFBP-2 and decreased IGFBP-3 and bone density increased

Grinspoon et al., 2003 [272]	65 W anorexia nervosa	25.6 ± 0.8	30 *μ*g/kg sc twice daily	9 months	—	—	IGF-I and IGFBP-3 are independent predictors of bone density

Misra et al., 2009 [273]	10 W low bone density and anorexia	12–18	30–40 mcg/k twice daily	7–9 days	↑	↑	Increase in PINP, a bone formation marker

M = men, W = women, PMO = postmenopausal women, MIO = men with idiopatic osteoporosis, BR = bone resorption, BF = bone formation.

**Table 3:** Effect of GH and IGF-1 administration on bone fractures healing.

Authors	Number of subjects	Age	Type of fracture	Therapy	Dose GH (mg/kg/day)	Duration	Results
Van der Lely et al., 2000 [274]	Placebo = 46 W = 42 M = 13	76.5 ± 7.2 23.6 ± 3.2	Hip fracture	rHGH	0.02	6 weeks	75% of patients return to the prefracture living situation

Boonen et al., 2002 [270]	9 M + 11 W	65–90	Hip fracture	rhIGF-I/IGFBP-3	0.5 = 9 1.0 = 11	8 weeks 6 months	Increase bone density and muscle strength and enhance functional recovery

Yeo et al., 2003 [275]	31 W	86 medium	Hip fracture	rHGH	0.05 (high dose) or 0.025 (low dose)	14 days	Significant increase of serum IGF-I and IGFBP-3 and promotes anabolism

Weissberger et al., 2003 [276]	33 W	60–82	Total hip replacement	rHGH	0.012	14 weeks preoperatively and 4 weeks postoperatively	Improvements in lean body mass and skeletal muscle mass

Hedström et al., 2004 [277]	20 W	<65	Hip fracture	rHGH	0.1 U max 8 U	4 weeks	IGF-I increased significantly and lean body mass and BMC preserved

Raschke et al., 2007 [278]	406 93 W + 313 M	18–64	Tibial fracture	rHGH	15, 30, or 60	16 weeks	GH did not accelerate time to healing in open fracture

Krusenstjerna-Hafstrøm et al., 2011 [279]	406 (313 males and 93 females)	56 ± 8.4	Tibial fracture	rHGH	15, 30, or 60	16 weeks	Dose-dependent increases of bone markers∗

M = men, W = women.
